# Multifunctional Magnetocontrollable Superwettable‐Microcilia Surface for Directional Droplet Manipulation

**DOI:** 10.1002/advs.201900834

**Published:** 2019-07-15

**Authors:** Shuang Ben, Tiantian Zhou, Han Ma, Jinjia Yao, Yuzhen Ning, Dongliang Tian, Kesong Liu, Lei Jiang

**Affiliations:** ^1^ Key Laboratory of Bio‐Inspired Smart Interfacial Science and Technology, School of Chemistry Beihang University Beijing 100191 P. R. China; ^2^ State Key Laboratory of Explosion Science and Technology Beijing Institute of Technology Beijing 100081 P. R. China; ^3^ Beijing Advanced Innovation Center for Biomedical Engineering Beihang University Beijing 100191 P. R. China

**Keywords:** directional transportation, liquid manipulation, magnetic microcilia array surface, superwettable, switchable structures

## Abstract

In nature, fluid manipulations are ubiquitous in organisms, and they are crucial for many of their vital activities. Therefore, this process has also attracted widescale research attention. However, despite significant advances in fluid transportation research over the past few decades, it is still hugely challenging to achieve efficient and nondestructive droplet transportation owing to contamination effects and controllability problems in liquid transportation applications. To this end, inspired by the motile microcilia of micro‐organisms, the superhydrophobicity of lotus leaves, the underwater superoleophobicity of filefish skin, and pigeons' migration behavior, a novel manipulation strategy is developed for droplets motion. Specifically, herein, a superwettable magnetic microcilia array surface with a structure that is switchable by an external magnetic field is constructed for droplet manipulation. It is found that under external magnetic fields, the superhydrophobic magnetic microcilia array surface can continuously and directionally manipulate the water droplets in air and that the underwater superoleophobic magnetic microcilia array surface can control the oil droplets underwater. This work demonstrates that the nondestructive droplet transportation mechanism can be used for liquid transportation, droplet reactions, and micropipeline transmission, thus opening up an avenue for practical applications of droplet manipulation using intelligent microstructure surfaces.

## 1 Introduction

The development of materials whose geometric shapes are responsive to external stimuli offers fundamental approaches for studying the relationship between the physical forces and the formed shapes and lays the foundation for advancing the development of driving technology‐related dynamic systems.^1^ Micro and nanostructured shape‐memory materials are some of the most important materials in such dynamic systems^2^ owing to their significance for droplet manipulation^3^ and liquid transportation.^4^ Over the past few decades, materials with anisotropic wettability or geometric gradients for droplet or fluid manipulation^5^ have received extensive attention both in basic research and for practical applications. In particular, micro and nanostructured materials that are affected by external fields have been shown to have many advantages.[qv: 3b,6] Specifically, magnetic surface structures that can be reversibly switched by exerting a changed magnetic field have advantages for transporting objects, such as real‐time control and a high positional accuracy.^7^ Accordingly, scientists have expended significant effort to develop a large number of versatile materials with magnetic interfaces, such as microciliated surfaces,^8^ magnetic oblique nanowire array surfaces,^9^ magnetic responsive film,^10^ magnetic elastic surfaces,^4^, ^11^ and ferrofluid or nanoarray complex interfaces.[qv: 1c,7c,12] However, it remains a challenge to achieve nondestructive, and efficient droplet transportation owing to contamination problems or the loss of liquid, and because of controllability problems in liquid transportation applications.

In nature, many micro‐organisms rely on motile cilia to control their direction of movement and for life‐sustaining activities.^13^ Further, the response of magnetite in a homing pigeons' body to the geomagnetic field assists them in orientation, long‐distance migration, and homing.^14^ Magnetically controllable artificial cilia may therefore provide impetus to drive the movement of a liquid droplet.^15^ To achieve nondestructive and efficient transportation of droplets, a smart responsive surface with low adhesion is highly sought‐after. Lotus leaves and filefish skin[qv: 8b,16] inspired “self‐cleaning” surfaces provide opportunities for designing advanced materials owing to their superwettability and low‐adhesion surface.^17^

Inspired by micro‐organisms' motile cilia, “self‐cleaning” lotus leaves, and pigeons' migration behaviors, herein, we demonstrate a strategy to manipulate directional water droplet movement on a superhydrophobic magnetic microcilia array (MMA) surface with low adhesion, which was prepared via template and a superhydrophobic nanosilica‐*n*‐hexane solution immersion method. The structure of the superhydrophobic MMA surface can be altered by applying an external magnetic field, allowing the water droplets to be manipulated effectively, continuously, and directionally. On the other hand, inspired by underwater superoleophobicity of filefish skin,[qv: 8b,16] we also design a strategy to manipulate oil droplet movement on an underwater superoleophobic magnetic microcilia array surface with low adhesion by applying an external magnetic field.^18^ Moreover, compared with the various magnetic response droplet manipulation techniques,^8^, ^9^, ^10^, ^11^, ^12^ this novel manipulation technique not only enables nondestructive, fast, precise, and reversible manipulation of droplets on a horizontal surface in air or underwater, but also can realize droplet manipulation on an inclined surface in air or underwater. This work provides a new avenue for research into droplet manipulation and liquid transportation; our approach has applications for droplet reactions and micropipeline transmission and expands the practical applications of microstructural intelligent surfaces.

## 2 Results and Discussion

### 2.1 Superhydrophobic MMA Surface

#### 2.1.1 Morphology and Infiltration Behaviors of the Prepared Superhydrophobic MMA Surface

**Figure 1:**
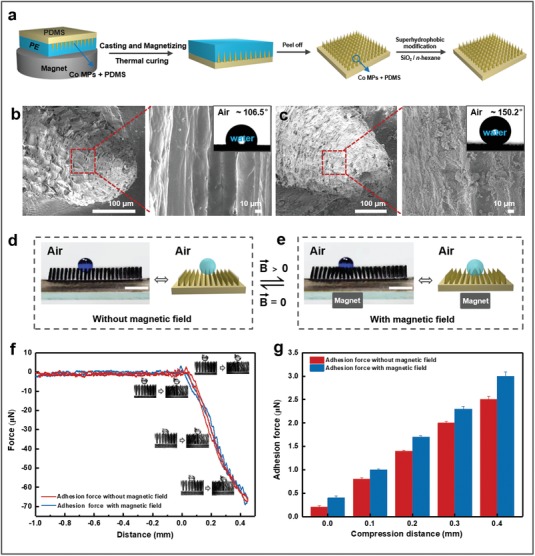
Fabrication of superhydrophobic magnetic microcilia array (superhydrophobic MMA) surface and droplet infiltration behavior with and without an applied magnetic field. a) Schematic of the fabrication procedure of the superhydrophobic MMA surface. b,c) Morphology and corresponding water droplet contact angle photographs of the magnetic microcilia before and after superhydrophobic modification, respectively. d,e) Switched water droplet infiltration states on the superhydrophobic MMA surface before and after the application of the magnetic field. When a magnet (0.37 T) was placed below the superhydrophobic MMA surface, the microcilia reshaped themselves from a fully upright to a concave cluster structure. Correspondingly, the water droplet sank into the microcilia. The scale bars represent 5 mm. f,g) Adhesion force–distance charts for the water droplets (*V* = 10 µL) on the superhydrophobic MMA surface before and after application of the magnetic field. The results show that the adhesion force between the microcilia and water droplets is low, indicating that the droplets can be easily moved into and out of the collapsed concave region, thus following the moving magnetic field. The droplets can advance easily because the superhydrophobic MMA surface is concave under the applied magnetic field. Furthermore, it is easy for the droplets to retreat, because the superhydrophobic MMA surface is a superhydrophobic and low‐adhesion surface.

To be able to control the transport direction of water droplets, an MMA surface was first fabricated using template technology;[qv: 17a] afterward, the surface was immersed in a solution of hydrophobic fumed nanosilica in *n*‐hexane to obtain a superhydrophobic MMA surface (**Figure** **1**a). By comparing the MMA surface morphologies before (Figure 1b) and after hydrophobic modification (Figure 1c), we demonstrate the existence of microscale structures on the MMA surface, while micro/nano hierarchical structures are formed on the superhydrophobic MMA surface. Based on the Cassie equation,^19^ surface hydrophobicity is enhanced owing to the increase in surface micro/nanoscale hierarchical structures, resulting in the MMA surface becoming superhydrophobic. Furthermore, in comparison with the conventional superhydrophobic modification technology, this method achieves more stable superhydrophobic structure on the MMA surface. Because of the swelling properties of polydimethylsiloxane (PDMS) in *n*‐hexane,^20^ the nanosilica in the mixed solution has been embedded in the MMA at its surface. As there are strong interactions between the nanosilica and PDMS, the mechanical stability of the superhydrophobic MMA surface is well preserved, even after scratching it with glass (Figure S1, Supporting Information).

The superhydrophobic MMA cilia are magnetically responsive owing to the addition of cobalt (Co) magnetic particles (MPs). Under an alternating external magnetic field, the surface structure alternates between a clustered and declustered structure, thus demonstrating reversible switching of the surface structure. As a result, when a water droplet is put on this superhydrophobic MMA surface, the microcilia are fully upright to prop‐up the water droplet (Figure 1d). In contrast, the microcilia transform from the fully upright to the clustered morphology when a magnet is placed below the superhydrophobic MMA surface (magnetic field intensity of about 0.37 T) (Figure 1e). Correspondingly, the water droplet sinks into the concave microcilia region. Enhancing the magnetic field results in only a small increase of the adhesion force between the superhydrophobic MMA surface and the water droplets (Figure 1f,g), indicating that the superhydrophobic MMA surface has low‐adhesion characteristics. As a result, it is easy to advance and retreat the droplets along the superhydrophobic MMA surface at the concave points under the magnetic field.

#### 2.1.2 Effect of Microcilia Size on Water Droplet Transportation

**Figure 2:**
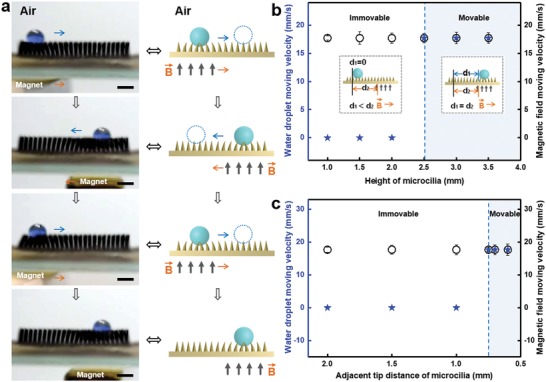
Horizontal motion characteristics of water droplets on the magnetic responsive superhydrophobic MMA surface. a) Optical images and schematic illustration of the droplet transport process on the superhydrophobic MMA surface under an applied external magnetic field. When the magnet moves to the right or left, the droplet follows the magnet to the right or left, respectively. The scale bars represent 5 mm. b) Effect of the superhydrophobic MMA height (*H*) on the movability of the water droplets (*V*_droplet_ = 12 µL) with the distance between adjacent microcilia tips (*l*) of 0.7 mm and a magnetic field intensity (*B*) of 11 mT. ⋆ and ⚪ represent water droplet moving velocity and magnetic field moving velocity, respectively. The insets are schematic drawings of the water droplet motion when the droplet does and does not follow the moving magnetic field (right of the blue line, representing “movable” and left of the blue line, “immovable,” respectively). Here, “*d*_1_” and “*d*_2_” indicate the distance the water droplet and the magnetic field have moved, respectively. c) Influence of the adjacent tip distance of the microcilia on the movability of the water droplets (*V*_droplet_ = 12 µL, *H* = 2.5 mm, and *B* = 11 mT). The results indicate that the water droplet can be driven on the superhydrophobic MMA surface by the external magnetic field when 2.5 mm ≤ *H* ≤ 4.0 mm and 0.5 mm ≤ *l* ≤ 0.7 mm.

As the superhydrophobic MMA surface morphology showed a good response to an applied external magnetic field, we also investigated the motion of a water droplet on the superhydrophobic MMA surface. When a magnet is brought close to the superhydrophobic MMA surface, a tiny concave region can be generated. The water droplets in the tiny concave region can be moved by controlling the location of the magnetic field (**Figure** **2**a). When the magnetic field moves to the right, the droplet follows the magnetic field to the right; and when the magnetic field moves in the opposite direction, the droplet also moves back under the action of this propulsive force. The result clearly demonstrates that a droplet can be moved on the superhydrophobic MMA surface by manipulating a magnetic field. Since this droplet transport relies on the microcilia array surface, we studied the effect of the microcilia properties on the transportation of water droplets. We find that the height of the microcilia array (*H*) is critical for the transportation of a water droplet. The results indicate that when *H* is smaller than 2.5 mm, the microcilia cannot transport the water droplet (Figure 2b; Figure S2, Supporting Information). Conversely, microcilia arrays with a height greater than 2.5 mm can efficiently achieve directional manipulation of a water droplet. This may be because of the tiny concave region generated through the ciliary curve; the driving force for the movement of the water droplet stems from this tiny concave region. Short microcilia exhibit a weak magnetic response,^15^, ^21^ resulting in a smaller curvature of the microcilia, i.e., the tiny concave region formed on the surface, and thus the driving force is too weak to push the water droplets. However, it is not true that the longer the microcilia, the better the transportation behavior. First, since there are some breakpoints in the soft microcilia with built‐in cobalt particles (Co MPs), it is difficult to peel off the complete ciliary array from the template when microcilia height is greater than 4.0 mm. Second, as the height of microcilia increases to more than 4.0 mm, the microcilia will collapse due to their softness. Consequently, the microcilia cannot support water droplets, causing the water droplets to sink into the microcilia array. The sinking behavior may lead to an increase of the contact area and adhesion force between the superhydrophobic MMA surface and water droplet. As a result, the manipulation cannot be achieved. Therefore, the height of the microcilia ranging from 2.5 to 4.0 mm is recommended. The distance between adjacent microcilia tips in the microcilia array (*l*) is also a very important factor in droplet transportation. When the volume of the water droplet is constant, the number of microcilia propping up the water droplet decreases as the adjacent tip distances of the microcilia are increased; when the distance is too large, the water droplet cannot be supported on the surface of the microcilia, instead sinking into the microcilia array or being punctured by it (Figure S3, Supporting Information). On the contrary, if the distance between the two cilia is smaller than 0.5 mm, much more overlap is generated while applying a magnetic field. Therefore, the microcilia can interfere with each other,^22^ resulting in smaller concave surfaces. Consequently, the microcilia cannot generate enough impact force for droplet motion. The results indicate that the surface can efficiently transport water droplets when 0.5 mm ≤ *l* ≤ 0.7 mm (Figure 2c), but cannot when *l* < 0.5 mm or *l* > 0.7 mm. Therefore, the water droplet can be driven along on the superhydrophobic MMA surface by an external magnetic field when 2.5 mm ≤ *H* ≤ 4.0 mm and 0.5 mm ≤ *l* ≤ 0.7 mm.

#### 2.1.3 Influence of Water Droplet Size on Water Droplet Transportation

**Figure 3:**
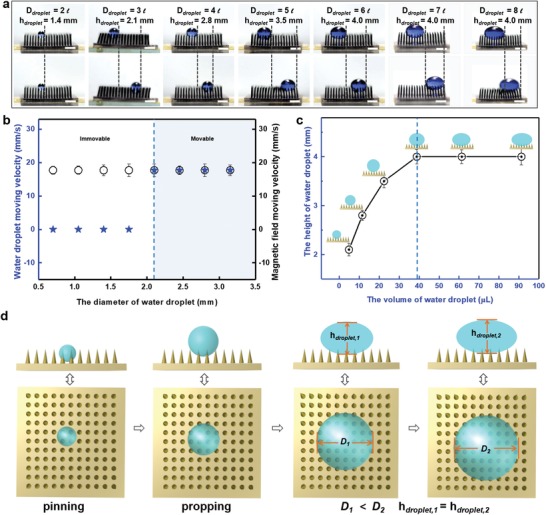
Magnetic field driven movability of water droplets with different sizes on the superhydrophobic MMA surface. a) The movability of water droplets with different sizes on the superhydrophobic MMA surface (*H* = 3.5 mm, *l* = 0.7 mm, and *B* = 11 mT). The scale bars represent 3.5 mm. b) When the diameter of the droplet was at least 3*l*, i.e., *D* ≥ 3*l*, the water droplets can be transported on the superhydrophobic MMA surface by the moving magnetic field. c) The height of the water droplet on the superhydrophobic MMA surface changes as the diameter of the droplet increases. When the volume of the droplet reaches a certain value, the droplet remains almost constant in height (*h*_droplet_ = 4.0 mm) on the superhydrophobic MMA surface. d) Illustration of the effect of the water droplet sizes on its shape and motion characteristics. When the droplet volume is gradually increased, the droplet gradually changes from the pinned state to the drivable state, but the droplet height does not change.

Based on the fact that the movement of the water droplets can only be achieved when the adjacent microcilia tip distance is less than 0.7 mm, the size of the droplets may also be important. We thus also investigated the effect of droplet size on transport performance. As shown in **Figure** **3**a,b, when the droplet diameter (*D*) is less than three times the adjacent microcilia tip distance (*D* < 2.1 mm), the droplet sinks and gets caught in the gap between the microcilia; as a result, the droplet cannot follow the movement of the magnetic field. Conversely, when the droplet diameter is equal to or greater than three times the adjacent microcilia tip distance (*D* ≥ 2.1 mm), controllable directional transport of the droplet can be achieved on the superhydrophobic MMA surface via a magnetic field. Furthermore, since the maximum volume of water that each cilium can prop up is constant, when this maximum is reached and the droplet volume continues to increase, the liquid spreads in the horizontal direction. Owing to the surface tension of the liquid,^23^ the droplets do not break up into several smaller water droplets; instead the water contact angle decreases and the height of the droplets (*h*_droplet_) remains almost constant as the droplet diameter increases to six times (*D* = 4.2 mm) the distance between adjacent ciliary tips (Figure 3c). Hence, no matter how great the droplet's volume is, liquid droplets can be directionally manipulated by using a large enough superhydrophobic MMA surface and an appropriate magnetic field.

#### 2.1.4 Influence of the Magnetic Field on Water Droplet Transportation

**Figure 4:**
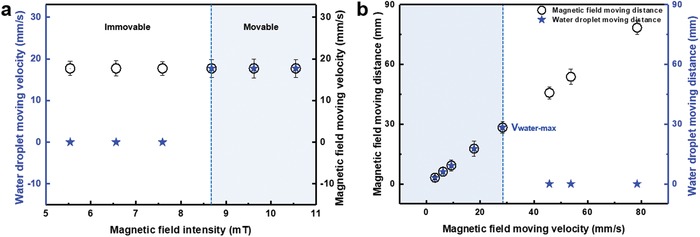
Water droplet motion characteristics on the superhydrophobic MMA surface as a function of the magnetic field intensity. a) The movability of the water droplet (*V*_droplet_ = 12 µL) on the superhydrophobic MMA surface (*l* = 0.7 mm and *H* = 3.5 mm) with different magnetic field intensities. b) The response velocity limit, i.e., the maximum velocity at which a water droplet can follow the motion of the magnetic field, is ≈28.3 mm s^−1^.

Since the water droplet's movement is dependent on the motion of the magnetic field, we investigate the influence of the magnetic field and its velocity on the water droplet's movement. When a water droplet (12 µL) is added onto the superhydrophobic MMA surface (*l* = 0.7 mm, *H* = 3.5 mm), the droplet exhibits different states of motion under different magnetic fields. Because the driving force results from the tiny concave region generated through a ciliary curve, applying a weak magnetic field reduces the degree of the microcilia curving, and the microcilia cannot generate sufficient impact force to move the water droplet. As shown in **Figure** **4**a, when the magnetic field intensity, *B*, at the water droplet's location is less than 8.7 mT (*B* < 8.7 mT), the droplet cannot follow the magnet's movement, whereas it is able to do so as long as *B* ≥ 8.7 mT. We also find that during droplet transport, droplets can follow the movement of the magnetic field (*B* ≥ 8.7 mT) when tuning the magnetic field velocity between 0 and 28.3 mm s^−1^. The maximum transport speed limit is 28.3 mm s^−1^ (Figure 4b). In previous studies, the direction and the speed of the droplet's motion depend on the wettability gradient^9^, ^24^ between the surface and the water droplet. However, in this work, the direction and speed of the droplet's motion are tuned by the switching of the surface's structure owing to a moving magnetic field, and the droplet's motion is not affected by the fixed wettability gradient.

#### 2.1.5 Demonstration of Water Droplet Manipulation on the MMA Surface

**Figure 5:**
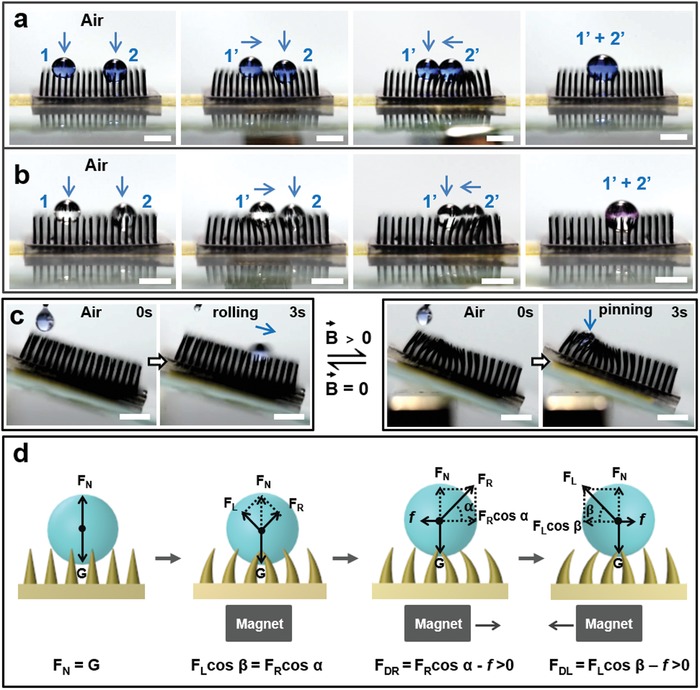
Mechanism underpinning aqueous liquid manipulation via magnetic response and demonstration thereof. a) Transportation of two droplets, followed by a merging and mixing process on a superhydrophobic MMA surface. In brief, one water droplet (1) on the left side is transported to the right side along the direction of the arrow, coming to rest in the middle (1′) of the superhydrophobic MMA surface. Then, another water droplet (2) on the right side moves leftward and approaches the first droplet (1′). Finally, water droplets (1) and (2) merge into one big droplet (1′ + 2′) and mix. b) A simple chemical reaction between one droplet of 0.1 m NaOH solution and another of diluted phenolphthalein indicator on the superhydrophobic MMA surface based on the above‐mentioned mixing process. The NaOH droplet (1) is transported along the direction of the arrow, and the phenolphthalein droplet (2) moves toward the NaOH droplet (1′) to merge into one pink droplet (1′ + 2′). This process confirms that positioning microscopic reactions can be realized on this surface. c) The droplets can be switched between states by changing the magnetic field: either rolling down or being pinned to the inclined superhydrophobic MMA surface. A water droplet was shown to slip on the superhydrophobic MMA surface at an incline angle of 15° when no magnetic field was applied; however, when a magnetic field of 0.37 T was applied, the water droplet did not slip on the same inclined superhydrophobic MMA surface. The scale bars represent 5 mm. d) Analysis of the mechanism of the water droplet movement process on the superhydrophobic MMA surface. A water droplet is placed on the superhydrophobic MMA surface with totally upright microcilia. When a magnetic field is applied directly below the superhydrophobic MMA surface, the microcilia bend inward from both sides toward the center of the magnetic field; this process is reversible when turning off the magnetic field. The water droplet reaches a force‐balanced state within the concave region formed on the surface. When the external magnetic field moves horizontally toward the right, the microcilia bend based on the location of the magnetic field. This results in unbalanced forces acting on the water droplet, which push the droplet to move. Correspondingly, when the external magnetic field is moved back to the left, the droplet also moves in an inequilibrium state.

To demonstrate the droplet manipulation properties of the superhydrophobic MMA surface, we investigated how droplets coalesced on it. As shown in **Figure** **5**a, two water droplets are placed on the surface. First, the water droplet on the left side (1) is transported to the right along the direction of the arrow, coming to rest in the middle (1′) of the superhydrophobic MMA surface. Then, the other water droplet (2) on the right side is moved toward the left and approached droplet (1′). Finally, water droplets (1) and (2) merge into one big droplet (1′ + 2′) and mix. We also performed a simple chemical reaction (Figure 5b). In brief, we created one droplet of 0.1 m NaOH solution and one of diluted phenolphthalein indicator and merged those droplets on the superhydrophobic MMA surface using the above‐mentioned process. It is found that the NaOH droplet (1) is transported along the direction of the arrow, and the phenolphthalein droplet (2) is moved toward the NaOH droplet (1′) to merge into one pink droplet (1′ + 2′). This confirms that microscopic positioning reactions can be realized on this surface.

Furthermore, water droplet manipulation is achieved not only on a horizontal surface, but also on an inclined surface with some tuning. When a water droplet is placed onto an inclined superhydrophobic MMA surface (angle of ≈15°) without a magnetic field, it quickly rolls down the incline (Figure 5c). However, the water droplet does not slip when a magnet generating a 0.37 T magnetic field is placed below the same superhydrophobic MMA surface. Thus, the state of the droplets can be switched between “rolling down” and “pinned to” the inclined superhydrophobic MMA surface by alternatingly applying and not applying the magnetic field owing to the concave region of the surface generated by applying the magnetic field.

#### 2.1.6 Water Droplet Manipulation Mechanism of the MMA Surface

To thoroughly understand the mechanism underpinning the manipulation of the water droplet on the superhydrophobic MMA surface during the movement process, we broke down the droplet transportation induced by the magnetically driven superhydrophobic MMA surface into two processes and illustrated them in Figure 5d. When a magnetic field is applied directly below the microcilia, the microcilia respond reversibly to the magnetic field, bending in from both sides toward the center of the magnetic field. Thus, an equilibrium state is established for the water droplet under the resulting forces. The horizontal force components can be expressed by the following Equation (1)

(1) $$F_{R}\cos\left(\alpha \right)=F_{L}\cos(\beta )$$

where *F*_R_ is the normal force on the right side of the water droplet and α is the tilt angle of *F*_R_ (0° ≤ α ≤ 90°); *F*_L_ is the normal force on the left side of the water droplet; *F*_R_cos(α) and *F*_L_cos(β) are the horizontal components of *F*_R_ and F_L_, respectively; β is the tilt angle of *F*_L_ (0° ≤ β ≤ 90°). Herein, *F*_R_ and *F*_L_ are equal to the noncontact force (*F*_M_) of a permanent magnet, which is described by Equation (2) as follows^25^

(2) $$F_{M}=\frac{\mu_{0}\delta^{2}}{4SB^{2}L^{2}}$$

where *µ*_0_ is the permeability of vacuum, *µ*_0_ = 4π·10^−7^ *N*/*A* ^2^; δ is the air gap; *S* is the surface area of the permanent magnet; *B* is the magnetic field intensity; *L* is the length of the magnetic path.

During the movement of the magnet to the right, the resistance differs between the forward and backward directions of the water droplet. The resulting force (*F*_DR_) can be evaluated by the following Equation (3)

(3) $$F_{\text{DR}}=F_{R}\cos\left(\alpha \right)-f>0$$

where *F*_DR_ is the resulting force and *f* is the horizontal friction. Under the exerted *F*_DR_, the water droplet will move to the right on the superhydrophobic MMA surface.

Correspondingly, when the external magnetic field is moved back (leftward), the droplet is also in an inequilibrium state. The resulting force (*F*_DL_) can be evaluated by the following Equation (4)

(4) $$F_{\text{DL}}=F_{L}\cos\left(\beta \right)-f>0$$

Under the exerted *F*_DL_, the water droplet will move leftward on the superhydrophobic MMA surface.

### 2.2 Underwater Superoleophobic MMA Surface

**Figure 6:**
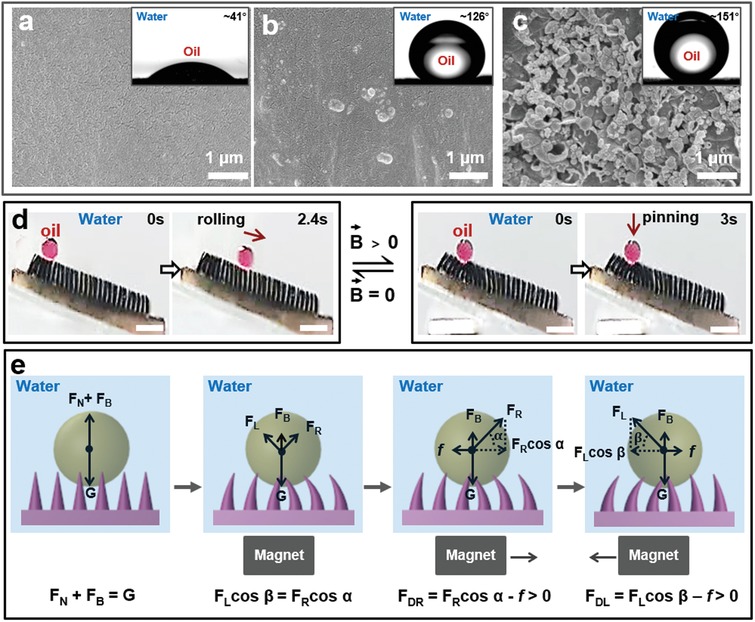
Mechanism underpinning oil liquid manipulation via magnetic response and demonstration thereof. a) Morphology and corresponding oil droplet contact angle photographs of the magnetic microcilia. b,c) Morphology and corresponding oil droplet contact angle photographs of the MMA surface modified with DA (2.0 mg mL^−1^) solution and the MMA surface modified with the mixture of DA (2.0 mg mL^−1^) and TEOS (6.0 mg mL^−1^) solution, respectively. d) The oil droplet manipulation was also achieved on an inclined surface (angle of ≈15°). The scale bars represent 5 mm. e) Analysis of the mechanism of the oil droplet movement process on the underwater superoleophobic MMA surface. When applying an external magnet, the oil droplet on the underwater superoleophobic MMA surface is subjected to the gravity (*G*), buoyancy (*F*_B_), and normal force (*F*_L_ and *F*_R_). The oil droplet reaches a force‐balanced state within the concave region formed on the surface. When the magnet moves, the oil droplet is subjected to friction (*f*), normal force (*F*_L_/*F*_R_), and buoyancy (*F*_B_). The forces in the vertical direction achieve equilibrium, while the forces in the horizontal direction are in a nonequilibrium state.

On the basis of the manipulation of aqueous droplets on superhydrophobic MMA surfaces, we studied the manipulation of oil droplets on the underwater superoleophobic MMA surfaces. Herein, we propose a novel and effective mussel‐inspired method^18^ for superoleophobic modification of PDMS microcilia. In brief, the prepared MMA surface was soaked in a mixture of dopamine (DA) and tetraethylorthosilicate (TEOS) with pH 8.5 to obtain an underwater superoleophobic MMA surface (**Figure** **6**; Figure S4, Supporting Information). Comparing the unmodified MMA surface with the MMA surface modified with DA (2.0 mg mL^−1^) solution (Figure 6b) and the MMA surface modified with the mixture of DA (2.0 mg mL^−1^) and TEOS (6.0 mg mL^−1^) solution (Figure 6c), we demonstrate that the MMA surface is successfully converted from underwater oleophilic to underwater superoleophobic after modification with the mixture of DA and TEOS. Because of the adhesive properties of polydopamine and poly(dimethylsiloxane),^18^ the reaction product nanosilica in the mixed solution has been adhered to the MMA surface. As there are strong interactions between the nanosilica and polydopamine and the strong interactions between the polydopamine and PDMS, the mechanical stability of the underwater superoleophobic MMA surface is well preserved, even after scratching it with glass (Figure S5, Supporting Information). Moreover, we have also accomplished the directional transport of oil droplets on the underwater superoleophobic MMA surface. We obtain the similar results as the water droplets transport on the superhydrophobic MMA surfaces, that the oil droplets can be driven on the underwater superoleophobic MMA surface by the external magnetic field (*B* ≥ 8.7 mT) when the adjacent microcilia tip distance in the microcilia array (*l*) is smaller than or equal to 0.7 mm (0.5≤ *l* ≤ 0.7 mm); the height of the microcilia array (*H*) ranges from 2.0 to 3.0 mm (2.0 mm ≤ *H* ≤ 3.0 mm); and the oil droplet diameter (*D*) is equal to or greater than three times the adjacent microcilia tip distance (*D* ≥ 2.1 mm) (Figure S6, Supporting Information). And the oil droplets can follow the movement of the magnetic field (*B* ≥ 8.7 mT) when tuning the magnetic field velocity between 0 and 31.5 mm s^−1^. The maximum transport speed limit for oil droplet is 31.5 mm s^−1^ (Figure S7, Supporting Information). Furthermore, the oil droplet manipulation can be achieved not only on a horizontal surface, but also on an inclined surface with some tuning (Figure 6d).

The transport mechanism of oil droplets on the underwater superoleophobic MMA surface is similar to that of water droplets on the superhydrophobic MMA surface. The difference is that the oil droplets are also subjected to buoyancy in water. Therefore, in the vertical direction, the sum of resultant force of buoyancy (*F*_B_) and normal force (*F*_N_) is equal to the oil droplet gravity (*G*) (Figure 6e). When applying an external magnet, the oil droplet on the underwater superoleophobic MMA surface is subjected to the *G*, *F*_B_, and normal force (*F*_L_ and *F*_R_). The oil droplet reaches a force‐balanced state within the concave region formed on the surface. When the magnet moves, the oil droplet is subjected to friction (*f*), normal force (*F*_L_/*F*_R_), and buoyancy (*F*_B_). The forces in the vertical direction achieve equilibrium, which can be described as the Equations (5) or (6)

(5) $$F_{R}\text{sin}\left(\alpha \right)+\;F_{B}=\;G$$

(6) $$F_{L}\text{sin}\left(\beta \right)+\;F_{B}=\;G$$

The resulting force in the horizontal direction can be evaluated by the Equations (3) or (4), where α is the tilt angle of *F*_R_ (0° ≤ α ≤ 90°); β is the tilt angle of *F*_L_ (0° ≤ β ≤ 90°); *F*_R_sin(α) and *F*_L_sin(β) are the vertical components of *F*_R_ and *F*_L_, respectively.

Consequently, the cleverly engineered surface with the superwettable MMA enables us to achieve directional transport of micro and nanometer‐scale droplets by adjusting the intensity and velocity of an applied magnetic field.

## 3 Conclusion

In conclusion, we implemented the bioinspired superwettable MMA surface for droplet transportation. Droplets on the superwettable MMA surface can be transported, continuously, in a specific direction under the motion of an external magnetic field. Moreover, the speed of the droplet motion can be adjusted by varying the magnetic field velocity. The superwettable MMA surface can thus be used as a pump to directly and continuously control the transport of liquids, e.g., the transport of aqueous liquids and 1,2‐dichloroethane, by modulating the applied magnetic field. In addition, we have achieved nondestructive and directional transportation of the droplets; the transport characteristics are affected by the properties of the microcilia and the speed of movement of the magnetic field, while not being limited by the droplet size. Thus, the proposed superwettable MMA surface provides a viable method to achieve directional transport of micro and nanometer‐scale droplets; it also provides a viable self‐cleaning mechanism. Our results have applications for future designs of droplet and microfluidic transportation devices.

## 4 Experimental Section

*Preparation of Magnetic Microcilia Array Surface*: The magnetic microcilia array surface was fabricated via a convenient template approach.^15^, ^21^ Briefly, a series of regularly arranged tapered holes were formed on a commercial polyethylene (PE) sheet by the impact of a mechanical arm on which a stainless steel needle was mounted. Cobalt magnetic particles (average diameter of 2 µm; Sigma‐Aldrich; PDMS prepolymer and Co MPs, weight ratio of ≈2.5:1) were then evenly dispersed into the tapered array holes. Then, an admixture composed of PDMS precursor (Dow Corning, SYLGARD 184, USA) and curing agent (Dow Corning, SYLGARD 184, USA) in a weight ratio of 10:1 was cast onto the template filled with the Co MPs. After that, the cast admixture was degassed for 2 h under vacuum with an Nd–Fe–B permanent magnet (about 0.7 T) placed below the template. Thereafter, the admixture was cured in an oven (80 °C, 6 h). After the thermal curing step, the sample was peeled off from PE.

*Preparation of Superhydrophobic Magnetic Microcilia Array Surface*: The as‐prepared microcilia array substrate was then soaked in a superhydrophobic SiO_2_ nanoparticle (average diameter of 14 nm, Evonik, Germany) solution (containing 0.1 g of superhydrophobic SiO_2_ nanoparticles and 40 mL of normal hexane) for 20 min. Then, the sample was taken out and placed in air for 24 h, and subsequently the superhydrophobic magnetic microcilia array surface was obtained.^21^, ^26^

*Preparation of Underwater Superoleophobic Magnetic Microcilia Array Surface*: Also, the as‐prepared microcilia array substrate was then soaked and stirred in a mixture of DA (2.0 mg mL^−1^, Macklin, China) and TEOS (6.0 mg mL^−1^, Aladdin, China) at pH 8.5 for 6 h. Then, the sample was taken out and stored in water, and subsequently the underwater superoleophobic magnetic microcilia array surface was obtained.[qv: 8b,16,18]

*Characterization and Instruments*: Environmental scanning electronic microscope images were collected using a JSM‐7500F environmental scanning electronic microscope (Japan). The behaviors of the droplets were captured by a digital camera (Powershot A1 1001S, Canon, Japan). Contact angles were measured using a Dataphysics OCA20 CA system. FT‐IR spectrum of magnetic microcilia with different treatment method was measured by Excalibur HE 3100 (Fourier, America). The adhesion force was measured by UpVideo DCAT.

## Conflict of Interest

The authors declare no conflict of interest.

## Supporting information

Supplementary
