# Supplementary material for: Multifunctional Magnetocontrollable Superwettable‐Microcilia Surface for Directional Droplet Manipulation
Source: Adv Sci (Weinh). 2019 Jul 15;6(17):1900834. doi: 10.1002/advs.201900834 (PMC6724473; doi:10.1002/advs.201900834)
Supplement: Supplementary file 1 — Supplementary [file ADVS-6-1900834-s001.pdf]

Copyright WILEY-VCH Verlag GmbH & Co. KGaA, 69469 Weinheim, Germany, 2019.

ADVANCED SCIENCE
Open Access

Supporting Information

for *Adv. Sci.,* DOI: 10.1002/advs.201900834

Multifunctional Magnetocontrollable Superwettable-Microcilia Surface for Directional Droplet Manipulation

Shuang Ben, Tiantian Zhou, Han Ma, Jinjia Yao, Yuzhen Ning, Dongliang Tian, Kesong Liu, and Lei Jiang*

WILEY-VCH

Copyright WILEY-VCH Verlag GmbH & Co. KGaA, 69469 Weinheim, Germany, 2019.

Supporting Information

Multifunctional Magnetocontrollable Superwettable-microcilia Surface for Droplets Directional Manipulation

Shuang Ben[†,‡], *Tiantian Zhou*[†,‡], *Han Ma*[˫], *Jinjia Yao*[‡], *Yuzhen Ning*[‡], *Dongliang Tian*[‡, §], *Kesong Liu*[‡, §,*], *and Lei Jiang*[‡, §]

†These authors contributed equally to this work.

Affiliations:

[‡] Key Laboratory of Bio-inspired Smart Interfacial Science and Technology of Ministry of Education, School of Chemistry, Beihang University, Beijing, 100191, P. R. China.

[§]Beijing Advanced Innovation Centre for Biomedical Engineering, Beihang University, Beijing 100191, China.

[˫]State Key Laboratory of Explosion Science and Technology, Beijing Institute of Technology, Beijing 100081, China

*Correspondence to:

Prof. Kesong Liu: E-mail: liuks@buaa.edu.cn

Supporting Note 1

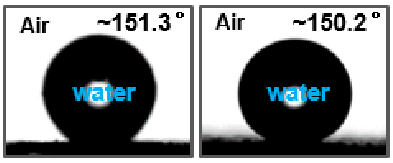

Figure S1. Contact angles of water droplet on the superhydrophobic MMA surface before and after scratching with glass, respectively. Owing to the strong interaction between nanosilica and PDMS, the mechanical stability of the superhydrophobic MMA surface is well preserved.

Supporting Note 2

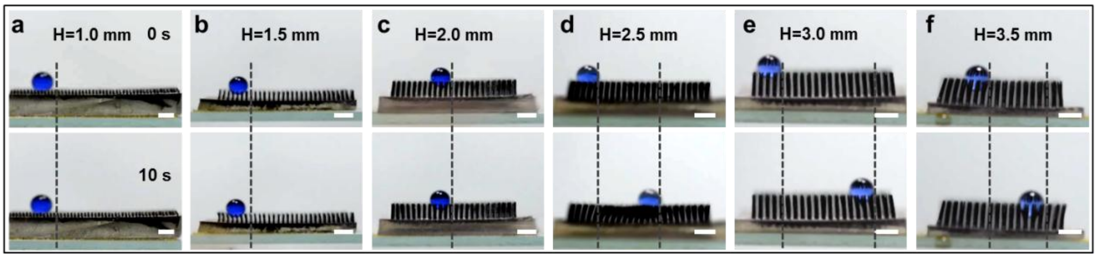

Figure S2. Optical photographs of water droplet manipulation with variations in magnetic microcilia height (*H*) under the condition of constant droplet volume ($V_{droplet}$ = 12 μL), adjacent tip distance of microcilia (*l* = 0.7 mm) and magnetic field intensity (*B* = 0.37 T). a-f) The heights of the microcilia are 1.0 mm, 1.5 mm, 2.0 mm, 2.5 mm, 3.0 mm and 3.5 mm, respectively. The result indicates that when the height of cilia arrays is smaller than 2.5 mm (i.e., $H < 2.5$ mm), they cannot transport water droplet. The scale bars represent 5 mm.

Supporting Note 3

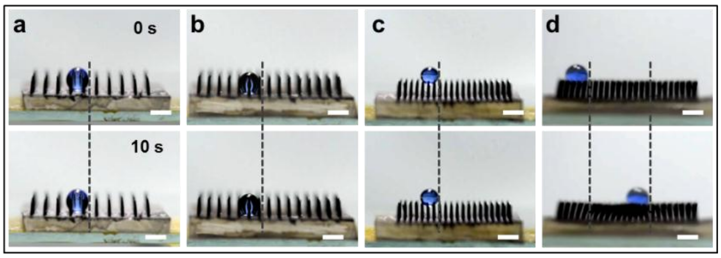

Figure S3. Optical photographs of water droplet manipulation with variations in adjacent tip distance of microcilia (*l*) under the condition of $V_{droplet}$ = 12 μL, *H* = 2.5 mm, *B* = 0.37 T. a-d) adjacent tip distances of the microcilia are 0.7 mm, 1.0 mm, 1.5 mm and 2.0 mm, respectively. The result shows that a separation distance between the adjacent tips of microcilia no more than 0.7 mm (i.e., $l \leq 0.7$ mm) can efficiently transport water droplet. The scale bars represent 5 mm.

Supporting Note 4

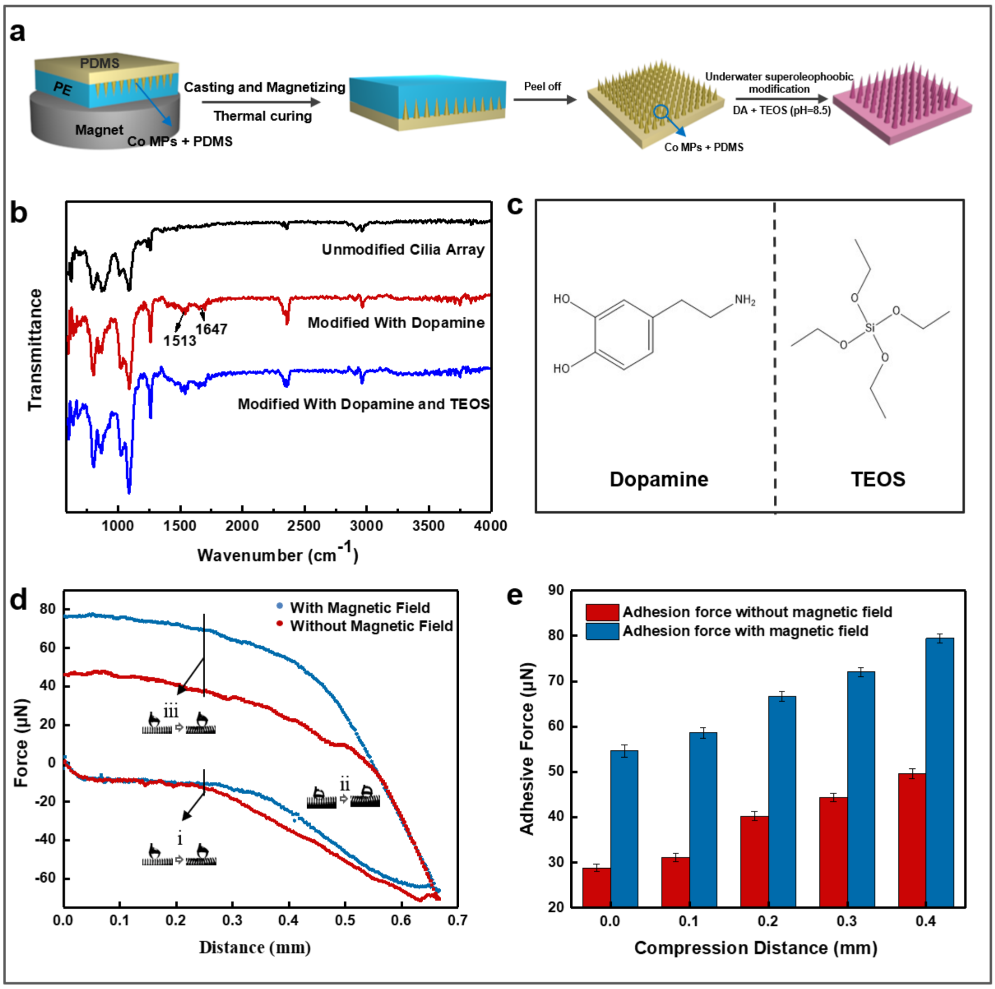

Figure S4. Schematic illustration for the fabrication of the underwater superoleophobic magnetic microcilia array (MMA) surface and corresponding conclusions. a) Schematic of the fabrication procedure of the underwater superoleophobic MMA surface. b) FT-IR spectrum of magnetic microcilia array with different treatment methods; as the spectrum depicts, compared to unmodified microcilia array, two absorption peaks appear at 1513 and 1647 cm^{-1}. We believe that the adsorption peak at 1647 cm^{-1} is caused by the overlap of C = C resonance vibration and N-H bending in the aromatic ring, and the N-H shear vibration of the amide group exists at 1513 cm^{-1}. Therefore, the polydopamine (*p*DA) coating is successfully adhered to magnetic microcilia array. c) Structural formula of DA and TEOS. d) Adhesion force-distance curves of the oil droplet (10 µL) and the cilia with and without magnetic field, respectively. The inset pictures are the oil droplets (V = 10 µL) on the underwater superoleophobic MMA surface before and after application of the magnetic field, respectively. e) Adhesion force between the underwater superoleophobic MMA surface and the oil droplet with and without magnetic field under different compression distance. When the compression distance is 0.4 mm, the adhesion forces between underwater superoleophobic MMA surface and oil droplet are nearly 45 µN without magnetic field and about 72 µN with the magnetic field, respectively. The adhesion force with the magnetic field is much higher than that without magnetic field, because the oil droplet has a larger contact area with underwater superoleophobic MMA surface when the magnetic field exists.

Supporting Note 5

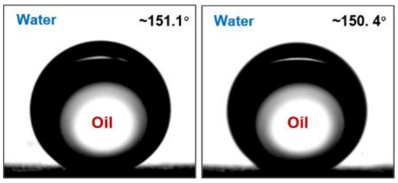

Figure S5. Contact angles of oil droplet on the underwater superoleophobic MMA surface before and after scratching with glass, respectively. Owing to the strong interactions between the nanosilica and polydopamine (pDA) and the strong interactions between the pDA and PDMS, the mechanical stability of the underwater superoleophobic MMA surface is well preserved.

Supporting Note 6

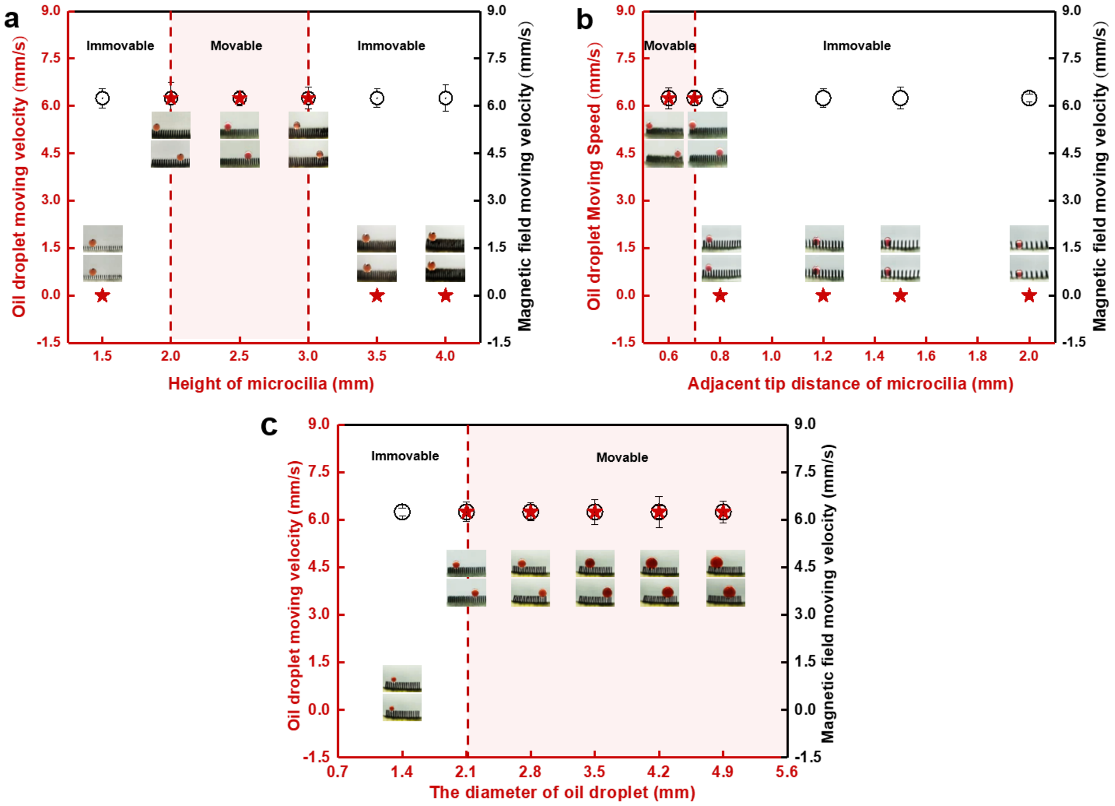

Figure S6. Effect of microcilia and oil sizes on oil droplet underwater manipulation. a) Influence of the underwater superoleophobic MMA height (*H*) on the movability of the oil droplets ($V_{droplet}$ = 12 μL) with the distance between adjacent microcilia tips (*l*) of 0.7 mm and a magnetic field intensity (*B*) of 11 mT. The insets are optical images of the oil droplet transport process on the underwater superoleophobic MMA surface under an applied external magnetic field. It is found that the height of the microcilia array (*H*) is critical for the transportation of an oil droplet. The results indicate that when *H* is smaller than 2.0 mm or higher than 3.0 mm, the microcilia cannot transport the oil droplet. In brief, microcilia arrays with a height in the range of 2.0 mm to 3.0 mm can efficiently achieve directional manipulation of an oil droplet. This may be because short microcilia exhibit a weak magnetic

response, resulting in a smaller curvature of the microcilia, i.e., the tiny concave region formed on the surface, and thus the driving force is too weak to push the oil droplet. However, as the height of cilia increases, the cilia may be not able to support oil droplet with the same size, causing the oil droplet to sink into the microcilia array. The sinking behavior leads to an increase of the contact area and adhesion force between the underwater superoleophobic MMA surface and oil droplet, so that the manipulation cannot be achieved. ★ and ○ represent oil droplet moving velocity and magnetic field moving velocity, respectively. b) Effect of the adjacent tip distance of the microcilia on the movability of the oil droplets ($V_{droplet}$ = 12 µL, H = 2.5 mm, and B = 11 mT). When the volume of the oil droplet is constant, the number of microcilia propping up the oil droplet decreases as the adjacent tip distance of the microcilia increases; when the adjacent tip distance is too large, the oil droplet cannot be supported on the surface of the microcilia, instead it sinks into the microcilia array or is punctured by it. The results indicate that the surface can efficiently transport oil droplets when 0.5 mm $\leq l \leq$ 0.7 mm, but cannot transport when l < 0.5 mm or l > 0.7 mm. c) When the diameter of the droplet is at least $3l$, i.e., $D \geq 3l$, the oil droplet can be transported on the underwater superoleophobic MMA surface by the moving magnetic field. When the droplet volume is gradually increased, the droplet gradually changes from the pinned state to the drivable state, as a result, the oil droplet could be manipulated. As a consequence, the oil droplet can be driven on the underwater superoleophobic MMA surface by the external magnetic field when 2.0 mm $\leq H \leq$ 3.0 mm, 0.5 mm $\leq l \leq$ 0.7 mm, and $D \geq$ 2.1 mm.

Supporting Note 7

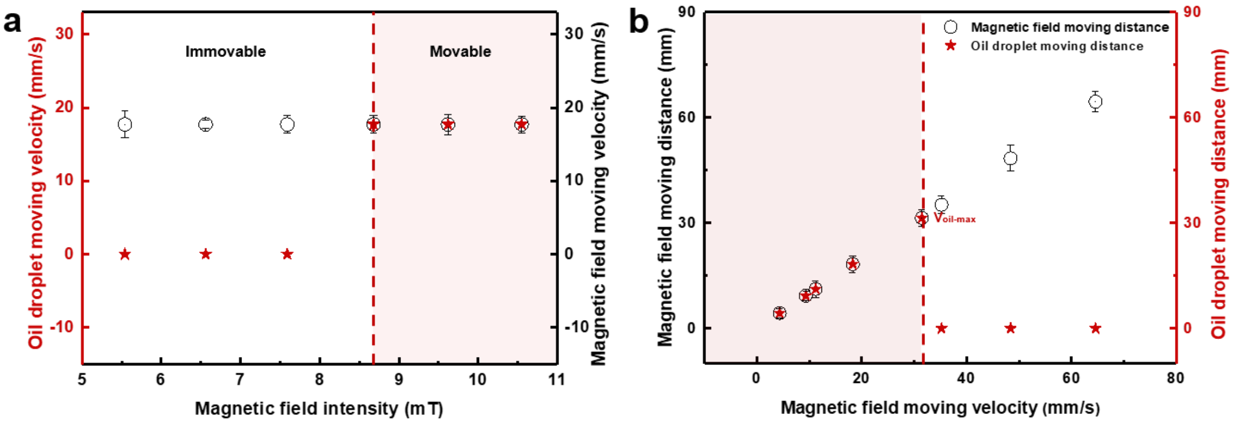

Figure S7. Oil droplet motion characteristics on the underwater superoleophobic MMA surface as a function of the magnetic field intensity. a) The movability of the oil droplet ($V_{droplet}$ = 12 µL) on the underwater superoleophobic MMA surface (l = 0.7 mm and H = 3.5 mm) with different magnetic field intensities. b) The response velocity limits, i.e., the maximum velocity at which an oil droplet can follow the motion of the magnetic field, is ~31.5 mm s^{-1}.
